# Negative Molecular Diagnostics in Non-Syndromic Hearing Loss: What Next?

**DOI:** 10.3390/genes14010105

**Published:** 2022-12-29

**Authors:** Thomas Clabout, Laurence Maes, Frederic Acke, Wim Wuyts, Kristof Van Schil, Paul Coucke, Sandra Janssens, Els De Leenheer

**Affiliations:** 1 Faculty of Medicine and Health Sciences, Ghent University, Corneel Heymanslaan 10, B-9000 Ghent, Belgium; 2 Department of Otorhinolaryngology, Ghent University Hospital, Corneel Heymanslaan 10, B-9000 Ghent, Belgium; 3 Center of Medical Genetics, Antwerp University Hospital and University of Antwerp, Prins Boudewijnlaan 43, B-2650 Edegem, Belgium; 4 Center for Medical Genetics, Ghent University, Corneel Heymanslaan 10, B-9000 Ghent, Belgium

**Keywords:** congenital hearing loss, deafness, molecular diagnostics, exome sequencing, *STRC* gene

## Abstract

Congenital hearing loss has an impact on almost every facet of life. In more than 50% of cases, a genetic cause can be identified. Currently, extensive genetic testing is available, although the etiology of some patients with obvious familial hearing loss remains unknown. We selected a cohort of mutation-negative patients to optimize the diagnostic yield for genetic hearing impairment. In this retrospective study, 21 patients (17 families) with negative molecular diagnostics for non-syndromic hearing loss (gene panel analysis) were included based on a positive family history with a similar type of hearing loss. Additional genetic testing was performed using a whole exome sequencing panel (WESHL panel v2.0) in four families with the strongest likelihood of genetic hearing impairment. In this cohort (*n* = 21), the severity of hearing loss was most commonly moderate (52%). Additional genetic testing revealed pathogenic copy number variants in the *STRC* gene in two families. In summary, regular re-evaluation of hearing loss patients with presumably genetic etiology after negative molecular diagnostics is recommended, as we might miss newly discovered deafness genes. The switch from gene panel analysis to whole exome sequencing or whole genome sequencing for the testing of congenital hearing loss seems promising.

## 1. Introduction

Hearing impairment is one of the most common sensory defects in children [1]. Based on neonatal hearing screening programs, permanent bilateral hearing loss is encountered in approximately 1.33 per 1000 live births [1,2,3]. Screening for congenital hearing loss should ideally be performed according to the 1-2-3 goal to limit developmental delay. This entails screening being completed by one month of age, whereas audiologic diagnosis should be completed by two months of age, and early intervention should not be initiated any later than three months of age [4].

The etiology of hearing loss is diverse. A genetic cause is presumed or identified in more than 50% of cases. About 25% of cases of congenital hearing loss are acquired, and less than 25% are idiopathic [5]. Although the hearing impairment of the majority of newborns with congenital hearing loss has a genetic etiology, 95% of them have hearing parents. Genetic cases can either be syndromic or non-syndromic. Hearing loss is syndromic when, apart from the hearing impairment, other clinical abnormalities are present, which is the case in 30% of patients. The other 70% of cases concern isolated deafness and are called non-syndromic hearing loss [6,7]. Acquired causes can be infectious or non-infectious, with congenital cytomegalovirus and rubella infections being the most prevalent, the latter of which are in a downward trend thanks to rubella vaccination programs [8,9,10]. Establishing an etiologic diagnosis of hearing loss is important, as it increases the degree of psychological well-being in patients and allows the physician to provide accurate information regarding recurrence risk, evolution and possible comorbidities. It also allows a better prediction of possible progression of the patient’s hearing loss [1,11].

The therapeutic options for hearing loss include conventional hearing aids, cochlear implants, and adapted educational needs. Conventional hearing aids are successfully used in most patients with mild to severe sensorineural hearing loss. However, for patients with severe to profound sensorineural hearing loss, a cochlear implant is usually preferred [3]. Finding the etiology of hearing loss can aid in choosing the most appropriate management options, as it usually results in a better understanding of the underlying physiopathology and the concomitant anatomical localization. This is especially important in the outcome of cochlear implants, as these bypass the membranous labyrinth but require a well-functioning auditory nerve and central auditory pathway to have good results. Mutations in genes preferentially expressed in the latter structures might thus be related to worse scores of cochlear implant performance than mutations preferentially expressed in the membranous labyrinth [12,13].

Given the prevalence of genetic hearing loss, molecular testing in an early stage is recommended. Technologic innovations in genetic research have expanded our knowledge on genetic hearing loss tremendously during the past decades. Where in early years single genes were tested sequentially, in present times a syndromic and/or non-syndromic test panel is widely implemented, whether or not it is preceded by *GJB2*/*GJB6* screening. Gene panels are regularly updated based on recent knowledge, and a transition from custom targeted panel testing to exome sequencing with a virtual panel has been introduced recently. Unfortunately, even after a comprehensive etiological work-up, the cause of hearing loss is not discovered in a considerable proportion of patients [5].

This article aimed to describe a cohort with negative molecular diagnostics for non-syndromic hearing loss with a strong likelihood of a genetic cause based on an obvious familial history for the same type of hearing loss. Furthermore, for some of those patients, we aimed to explain why no etiological diagnosis was found, and proved our hypotheses by additional genetic analyses. In addition, the management and future possibilities for genetic testing of patients with negative molecular diagnostics for non-syndromic hearing loss will be discussed.

## 2. Materials and Methods

A combination of a retrospective study and literature study was performed. Additional testing with an updated gene panel was performed in some patients after approval of the respective families. The study was approved by the Ethical Committee of Ghent University Hospital, Belgium.

### 2.1. Inclusion of Patients

Patients included in this article (*n* = 21) have been selected from the database of the otogenetics consultation of the otorhinolaryngology department of Ghent University Hospital, Belgium. Patients in whom no pathogenic mutation had been identified by a previous molecular analysis (gene panel analysis) were selected by a group of otorhinolaryngologists and geneticists of Ghent University Hospital based on a very high likelihood of having a genetic cause for their hearing loss. This likelihood was mainly based on an obvious familial history for the same type of hearing loss. Patients with arguments for a non-genetic cause of hearing loss (TORCHes infections, perinatal and postnatal risk factors) were excluded.

Based on the highest suspicion of genetic hearing loss and on their audiograms, which showed moderate to moderately severe hearing loss, eight patients of four families were contacted for additional genetic testing (whole exome sequencing), of whom seven agreed.

### 2.2. Mutation Analysis

All of the 21 included patients underwent genetic testing using the targeted gene panel for non-syndromic hearing loss at the Center for Medical Genetics, Antwerp, Belgium. In earlier years, this test was preceded by the exclusion of mutations in the *GJB2*/*GJB6* genes using Sanger sequencing by the Center for Medical Genetics Ghent, Belgium. A gene panel analysis was performed by SBS sequencing technology (Illumina, San Diego, CA, USA) after Haloplex enrichment of a gene panel of genes causing hearing loss. Different versions of the non-syndromic deafness gene panel (DOOF_v5_NS—DOOF_v11_NS, ranging from 87 to 115 genes) have been used as panel testing for those patients in the past. The retrieved variants were reported based on five classes depending on their likelihood to be pathogenic according to the recommendations of the American College of Medical Genetics and Genomics (ACMG) and the Association for Molecular Pathology (AMP) [14]. All patients were counselled during an otogenetic consultation.

Of the included patients, seven underwent whole exome sequencing conducted via SBS sequencing technology (Illumina, San Diego, CA, USA) after enrichment with the Twist Human Core Exome kit with additional human RefSeq transcripts and the mitochondrial genome (Twist Bioscience, South San Francisco, CA, USA). The 146 genes included in the WESHL panel v2.0 were analyzed for variants with JSI SeqPilot software v5.3.3 (Ettenheim, Germany) (Table A1). In addition, exome-wide HPO based filtering using MOON software (Diploid/Invitae, San Fransisco, USA) was performed. Variants in the *STRC* gene were confirmed via *STRC*-specific long-range PCR followed by a sequence analysis of the relevant *STRC* coding exons. Analysis for *STRC* copy number variants was performed using sequencing data and copy number loss was confirmed by multiplex ligation-dependent probe amplification (MLPA) analysis with the P461-A1kit (MRC-Holland, Amsterdam, The Netherlands).

Sequence data were analyzed with SeqNext analysis software (JSI Medical Systems, Ettenheim, Germany) against the Hg19 exome build reference sequence. For all individual genes a minimal 30× coverage was obtained for more than 95% of the coding sequences, and for the total gene panel a minimal 30× coverage was obtained for more than 98% of the coding sequences. A minimal minor allele frequency threshold of 15% was used for variant detection.

### 2.3. Database Preparation and Statistical Analysis

After the selection of patients, a database was created in Microsoft Excel (Microsoft, Redmond, WA, USA). This database included general information about the patients (age, sex), data on the type and etiology of hearing loss, severity, onset, type, symmetry and audiometric configuration of hearing loss, familial history of hearing loss, cytomegalovirus infection status, and results of molecular testing with the non-syndromic deafness gene panel of the Center for Medical Genetics Antwerp (DOOF_v5_NS—DOOF_V11_NS).

These data were obtained from the electronic health record of the patients. Severity of hearing loss was classified into six categories ((slight (16–25 decibel hearing level (dB)), mild (26–40 dB), moderate (41–55 dB), moderately severe (56–70 dB), severe (71–90 dB) or profound (≥90 dB)) [12]. For asymmetric hearing loss, the severity was classified based on the amount of hearing loss of the worst hearing ear. Figures were created using Microsoft Excel (Microsoft, Redmond, WA (USA)), Microsoft Visio (Microsoft, Redmond, WA (USA)), and BioRender.com (BioRender, Toronto, ON, Canada).

### 2.4. Literature Study

Different databases (PubMed, Google Scholar, Embase, Web of Science) were used to find relevant publications. The reference list of the most important publications was used to search for essential missing publications. EndNote 20 (Clarivate, London, UK) was used as the citation manager.

## 3. Results

### 3.1. Study Population

**Figure 1:**
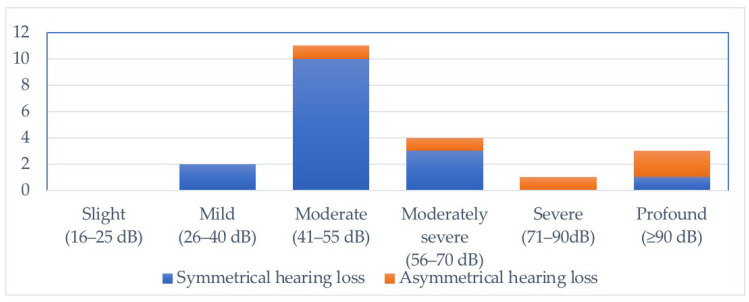
Severity of hearing loss for the included patients.

The selected study population included 21 patients, of whom 16 were male and 5 were female. Their ages at inclusion ranged between 4 and 13 years old, with the majority born between 2015 and 2018 (15 patients). All included patients had bilateral hearing loss. Fifteen of them had symmetrical hearing loss, whereas six had asymmetrical hearing loss. The hearing loss severity of the included patients can be found in Figure 1. The majority of patients demonstrate moderate hearing loss, followed by moderately severe hearing loss.

The targeted gene panel for non-syndromic hearing loss, performed in all patients, resulted in a total of 65 variants in 39 different genes (Table 1 and Table A2). Nine of these variants have an autosomal dominant pattern of inheritance, 47 have an autosomal recessive pattern, and nine variants are situated in genes with both autosomal dominant and recessive patterns of inheritance. However, all patients inherited the sequence variants found after genetic analysis heterozygously. In addition, all but two of these variants were classified as class 3. The other two variants were classified as class 4 and 5 variants, but as they were detected in combination with a class 3 variant, they did not (yet) explain the hearing loss. The gene panels used for each patient can be found in Table A2.

### 3.2. Additional Genetic Testing

**Figure 2:**
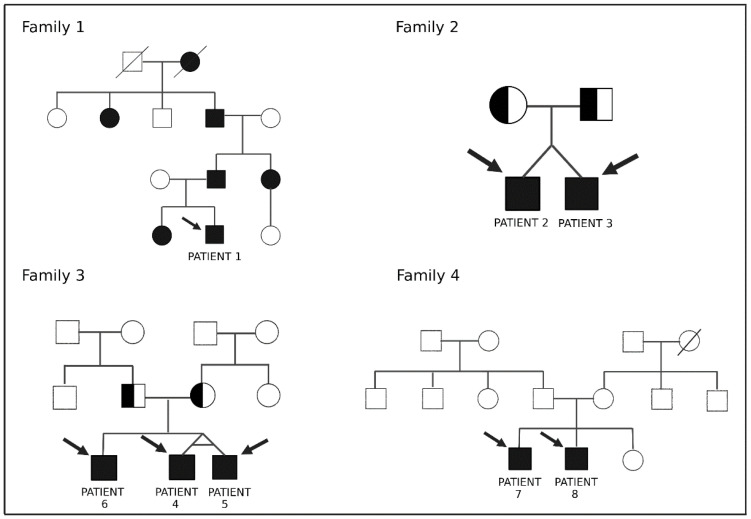
Pedigrees of the four selected families; circles are female and squares are male individuals, black icons are patients affected by hearing loss typical for the family, icons crossed by a line indicate deceased family members, arrows indicate patients included in the study.

Eight patients out of four families were selected for additional genetic testing based on the strongest familial history for the same type of hearing loss. Seven of them agreed to perform additional testing. The pedigrees of the four families are depicted in Figure 2, whereas Table 2 shows the results of the additional whole exome sequencing-based panel testing performed in these seven patients.

## 4. Discussion

In this study, patients with presumable hereditary hearing loss and negative molecular testing in the past have been investigated. We found most patients exhibiting moderate and moderately severe hearing loss (71%). Patients with profound hearing loss seem underrepresented compared with the general distribution of congenital hearing loss severity. In general, in more severe forms of hearing impairment the cause is more frequently found than in milder degrees of hearing loss [13]. This suggests that a higher severity of hearing loss is a positive predictor for identifying an underlying etiology. However, we should be careful with the hypothesis of patients with more moderate hearing loss being less likely to have genetic hearing loss. A more obvious explanation is that genes resulting in moderate hearing loss still need to be discovered.

Asymmetric hearing loss was present in 24% of our cohort. Sloan-Heggen et al. [15] reported that making an etiological diagnosis in patients with asymmetrical hearing loss is less frequent compared to patients with symmetrical hearing loss. However, the likelihood of a causative molecular defect is still higher for asymmetrical hearing loss compared to unilateral hearing loss.

Genetic variants were found in more than 40 different genes in the patients of the study cohort. To date, more than 120 genes are identified as causing non-syndromic hearing loss [16]. Custom targeted gene panels are modified according to the latest knowledge, but some of the included patients were tested years ago and were consequently not tested for all deafness genes known today. The gene panels used for each patient can be found in Table A1.

Of the included patients, 52% presented with moderate sensorineural hearing loss. The *STRC* gene has been shown to be the most commonly mutated gene in patients with this type of hearing impairment. *STRC* causes hearing loss in an autosomal recessive manner [15,17]. The *STRC* gene sequence data are difficult to interpret due to the existence of an almost identical pseudogene pSTRC [18]. The *STRC* gene was only recently (March 2020) added to the non-syndromic hearing loss panel used in the center for Medical Genetics in Antwerp. Based on a strong family history of hearing loss and the audiograms showing moderate to moderately severe hearing loss, a subset of patients with no molecular diagnosis was recontacted to perform an updated deafness gene panel containing the most recent deafness genes. More specifically, eight patients of four different families were recontacted, of whom seven agreed to participate. The main goal was to identify the molecular causes of hearing loss in additional deafness genes, and in particular in the recently added *STRC* gene. In half of the families (four out of seven patients), *STRC* pathogenic variants were found, some in cis with a *CATSPER2* deletion. The latter is a gene accounting for sperm motility. Deletions in this gene often go hand in hand with deletions in the *STRC* gene [19]. This genotype causes deafness-infertility syndrome (DIS), which is characterized by early-onset hearing loss in both male and female patients. In addition, the affected male patients are infertile. This is important in counseling the patients and their parents [19,20].

In addition to the detected disease-causing variants, we observed variants of uncertain significance (VUS) in several deafness genes. Variants in the *CDH23*, *MYO15A* and *PTPRQ* genes were mainly detected. Given the existence of digenic inheritance, it does not imply that a heterozygous variant in an autosomal recessive deafness gene is not involved in hearing loss. True digenic inheritance occurs when two non-allelic mutations on two separate genes are necessary and sufficient to cause disease [21]. Digenic inheritance of variants in the *CDH23* and *ATP2B2* genes and of variants in the *SLC26A4* gene and *FOXI10* or *KCNJ10* genes has been suggested [22,23,24]. However, a study of Landa et al. could not prove the latter [25]. In general, the evidence for digenic inheritance for hearing loss is still weak [11], and this mechanism was only suggested for combinations of genes not present in our study population.

With these results in mind, we see several opportunities to improve the diagnostic yield for genetic hearing impairment. Different genetic testing strategies can be used to detect genetic alterations that can cause hearing loss. Currently, next generation sequencing custom targeted gene panel testing is the gold standard for the genetic analysis of hearing loss in most centers. There are other ways to establish an etiological diagnosis, however. Three commonly used testing strategies (custom targeted next-generation sequencing panel-based testing, whole exome sequencing and whole genome sequencing) all have their own advantages and disadvantages, which we summarized in Table 3 [26,27,28,29,30,31]. Partly based on these evolutions, we recommend re-evaluating patients with unidentified hearing loss on a regular basis, in addition to the more frequent audiological follow-up. In this way, recent knowledge about novel deafness genes, modified variant calling and eventual digenic inheritance can be considered.

Initially, custom targeted gene panel testing was performed in this study population (*n* = 21). One of the largest disadvantages of this testing strategy is that only known deafness genes are included and that it is very static, as it is not easy to change these panels. A transition towards exome or even genome sequencing is becoming the gold standard. Exome sequencing with the use of virtual panels to restrict the analysis to specific genes related to a specific disorder using bioinformatic filtering is an increasingly favored approach for genetic testing. This technique has several advantages compared to the targeted approach. First, there is less chance of secondary findings compared to exome or genome sequencing without a virtual panel, thanks to the fact that only a panel of genes associated with hearing loss is analyzed. It also leads to less detection of variants with uncertain significance, which are often difficult to interpret and can cause uncertainty for both patients and clinicians. The technique is very flexible because genes can easily be added to and removed from the panel when new genetic knowledge becomes available. Even a retrospective analysis of novel deafness genes is possible without new blood sampling, again stressing the importance of the regular re-evaluation of patients [26,28,29,32].

Genetic variants are mostly classified into five classes based on the criteria of the ACMG-AMP [14]. In recent years, next-generation sequencing has enabled the performance of genetic tests on a large scale, providing ample genomic data. In addition to population data, computational and functional tools evolve, making more accurate variant classification possible [33,34,35]. To the best of our knowledge, no study has been performed to establish the reclassification rate in a population of patients who underwent genetic testing due to hearing loss. Such a study can be useful to establish whether variant reclassification is common for hearing loss.

This study has a few limitations. First, the study population only included 21 patients. In addition, the study is retrospective in design. There is also a selection bias because patients were not randomly selected, but were selected by an expert committee to be the most likely having genetic hearing loss. Minor information bias is also possible as the database is based on the patients’ electronic health records and different caregivers sometimes have a different way of interpreting clinical information.

## 5. Conclusions

In summary, clinical and audiometric re-evaluation combined with updated genetic testing can be successful in establishing an etiological diagnosis in some cases without a molecular diagnosis at first. The implementation of whole exome or whole genome sequencing with a virtual panel as the gold standard for genetic testing in hearing loss should be considered, instead of custom targeted gene panel testing. *STRC* seems to be a prevalent cause of hearing loss. In patients with previous negative molecular diagnostics for non-syndromic, mild to moderately severe hearing loss, the *STRC* gene should be analysed in case it was not performed in the past.

## Figures and Tables

**Table 1:** Variants found after initial genetic testing (AD = autosomal dominant, AR = autosomal recessive).

Gene	Mode of Inheritance	Number of Found Variants in Each Gene	Class of Found Variants	Homo- or Heterozygous Occurrence
*GJB2* *OTOGL* *SLC26A4* *LOXHD1* *THRAP3* *TECTA* *TBC1D24* *ATP6V0A4* *COL2A1* *CDH23* *MYO7A* *MYO15A* *OTOF* *GRXCR2* *PTPRQ* *TSPEAR* *ADCY1* *PJVK* *OTOA* *TRIOBP* *COCH* *RIPOR2* *WFS1* *MYO1A* *CLIC5* *COL11A2* *MTAP* *MYH14* *MYO3A* *LRTOMT* *USH2A* *MYH9* *GSDME* *DMXL2* *RDX* *GIPC3* *OTOG* *SLC17A8* *BDP1*	AR/AD AR AR AR AD AR/AD AR/AD AR AD AR AR/AD AR AR AR AR AR AR AR AR AR AR/AD AR AR/AD AD AR AR/AD AR AD AR AR AR AD AD AD AR AR AR AD AR	1 3 2 1 1 2 3 1 1 5 1 5 3 1 4 1 1 1 3 3 2 1 1 1 1 1 1 1 1 1 1 1 1 1 1 2 1 1 2	3 3 3,5 3 3 3 3 3 3 3 3 3 3 3 3 3 3 3 3 3 3 3 3 3 3 3 3 3 3 3 3 3 3 3 3 3,4 3 3 3	Heterozygous Heterozygous Heterozygous Heterozygous Heterozygous Heterozygous Heterozygous Heterozygous Heterozygous Heterozygous Heterozygous Heterozygous Heterozygous Heterozygous Heterozygous Heterozygous Heterozygous Heterozygous Heterozygous Heterozygous Heterozygous Heterozygous Heterozygous Heterozygous Heterozygous Heterozygous Heterozygous Heterozygous Heterozygous Heterozygous Heterozygous Heterozygous Heterozygous Heterozygous Heterozygous Heterozygous Heterozygous Heterozygous Heterozygous

**Table 2:** Results of additional whole exome sequencing-based panel testing.

Patient	Severity of Hearing Loss	Results of Additional Panel Testing
Family 1		
1	Moderately severe	Heterozygous *DMXL2* c.4937G > A (Class 3)
Family 2		
2	Moderate	Homozygous *STRC* and *CATSPER 2* deletion (Class 5)
3	Moderate	Homozygous *STRC* and *CATSPER 2* deletion (Class 5)
Family 3		
4	Moderate	Heterozygous *STRC* c.1030C > T (p.Arg344Ter) mutation and heterozygous *STRC* deletion (class 5)
5	Moderate	Heterozygous *STRC* c.1030C > T (p.Arg344Ter) mutation and heterozygous *STRC* deletion (class 5)
6	Moderate	Not tested
Family 4		
7	Moderately severe	Heterozygous *GIPC3* c.226-1G > T (already known, but now classified as class 5), recessive inheritance thus not considered responsible for the phenotype
8	Moderate	No variants found

**Table 3:** Advantages and disadvantages of custom targeted gene panels, whole exome sequencing and whole genome sequencing (VUS = variants of uncertain significance).

	Advantages	Disadvantages
Custom targeted gene panel testing	– Less VUS – Less secondary findings – Lower costs – Streamlined data analysis – Shorter turnaround time	– Static, quickly outdated – Only variants in known genes are detected
Whole exome sequencing	– Less selection bias – More flexibility in updating gene content if a panel is used – Reanalysis possible	– Higher cost (although plunging) – Defects in mitochondrial – DNA not routinely tested – Secondary findings – More VUS
Whole genome sequencing	– Better identification of large structural re- – arrangements, balanced translocations, uniparental isodisomy and mosaicism – The most unbiased sequencing method – Sequencing coding and non-coding regions – Better detection of copy number variants	– Higher cost – More VUS – Large amounts of data – More secondary findings

## Data Availability

All included data can be provided upon simple request.

## Appendix A

**Table A1:** Genes included in the exome sequencing analysis, together with their mode of inheritance and reference sequence (AD = autosomal dominant, AR = autosomal recessive).

Gene	Mode of Inheritance	Reference Sequence (GenBank)	Reference Sequence (Ensembl)
*ACTG1*	AD	NM_001199954.2	ENST00000331925
*ADCY1*	AR	NM_021116.4	ENST00000292723
*ADGRV1*	AR	NM_032119.4	ENST00000405460
*ATP6V0A4*	AR	NM_020632.3	ENST00000310018
*ATP6V1B1*	AR	NM_001692.4	ENST00000234396
*BSND*	AR	NM_057176.3	ENST00000371265
*CABP2*	AR	NM_016366.3	ENST00000294288
*CACNA1D*	AR	NM_00720.4	ENST00000288139
*CCDC50*	AD	NM_178335.3	ENST00000392456
*CD164*	AD	NM_006016.6	ENST00000310786
*CDC14A*	AR	NM_033312.2	ENST00000361544
*CDH23*	AR	NM_022124.6	ENST00000224721
*CEACAM16*	AD, AR	NM_001039213.4	ENST00000405314
*CHD7*	AD	NM_017780.4	ENST00000423902
*CIB2*	AR	NM_006383.4	ENST00000258930
*CISD2*	AR	NM_001008388.5	ENST00000273986
*CLDN14*	AR	NM_144492.3	ENST00000399137
*CLIC5*	AR	NM_001114086.2	ENST00000339561
*CLPP*	AR	NM_006012.4	ENST00000245816
*CLRN1*	AR	NM_174878.3	ENST00000327047
*COCH*	AD, AR	NM_004086.3	ENST00000396618
*COL11A1*	AD	NM_080629.2	ENST00000370096
*COL11A2*	AD, AR	NM_080680.3	ENST00000341947
*COL2A1*	AD	NM_001844.5	ENST00000380518
*COL4A3*	AD, AR	NM_000091.5	ENST00000396578
*COL4A4*	AD, AR	NM_000092.4	ENST00000396625
*COL4A5*	X-linked	NM_033380.3	ENST00000328300
*COL9A1*	AR	NM_001851.5	ENST00000357250
*COL9A2*	AR	NM_001852.4	ENST00000372748
*COL9A3*	AR	NM_001853.4	ENST00000343916
*DCDC2*	AR	NM_016356.5	ENST00000378454
*DIABLO*	AD	NM_019887.6	ENST00000443649
*DIAPH1*	AD	NM_005219.5	ENST00000398557
*DMXL2*	AD	NM_001174116.2	ENST00000543779
*EDN3*	AD, AR	NM_207034.3	ENST00000337938
*EDNRB*	AD, AR	NM_001201397.1	ENST00000377211
*ELMOD3*	AR	NM_001329793.2	ENST00000315658
*EPS8*	AR	NM_004447.6	ENST00000281172
*EPS8L2*	AR	NM_022772.4	ENST00000318562
*ERAL1*	AR	NM_005702.4	ENST00000254928
*ESRP1*	AR	NM_017697.4	ENST00000433389
*ESRRB*	AR	NM_004452.3	ENST00000380887
*EYA1*	AD	NM_000503.6	ENST00000340726
*EYA4*	AD	NM_004100.5	ENST00000367895
*FOXI1*	AR	NM_012188.5	ENST00000306268
*GIPC3*	AR	NM_133261.3	ENST00000322315
*GJB2*	AD, AR	NM_004004.6	ENST00000382848
*GJB3*	AD, AR	NM_024009.3	ENST00000373366
*GPSM2*	AR	NM_013296.5	ENST00000406462
*GRHL2*	AD	NM_024915.3	ENST00000251808
*GRXCR1*	AR	NM_001080476.2	ENST00000399770
*GRXCR2*	AR	NM_001080516.1	ENST00000377976
*GSDME*	AD	NM_004403.3	ENST00000342947
*HARS1*	AR	NM_002109.6	ENST00000504156
*HARS2*	AR	NM_012208.4	ENST00000230771
*HECTD3*	AR	NM_024602.6	ENST00000372172
*HGF*	AR	NM_000601.6	ENST00000222390
*HOMER2*	AD	NM_199330.3	ENST00000304231
*HSD17B4*	AR	NM_170743.4	ENST00000504811
*IFNLR1*	AD	NM_001199291.3	ENST00000327535
*ILDR1*	AR	NM_001199799.1	ENST00000344209
*KARS1*	AR	NM_001130089.1	ENST00000319410
*KCNE1*	AR	NM_000219.6	ENST00000337385
*KCNJ10*	AR	NM_002241.5	ENST00000368089
*KCNQ1*	AR	NM_000218.3	ENST00000155840
*KCNQ4*	AD	NM_004700.4	ENST00000347132
*KITLG*	AD, AR	NM_000899.5	ENST00000228280
*LARS2*	AR	NM_015340.4	ENST00000415258
*LHFPL5*	AR	NM_182548.4	ENST00000360215
*LOXHD1*	AR	NM_144612.6	ENST00000536736
*LRTOMT*	AR	NM_001145309.3	ENST00000435085
*MARVELD2*	AR	NM_001038603.3	ENST00000325631
*MCM2*	AD	NM_004526.4	ENST00000265056
*MIR96*	AD	NR_029512	ENST00000362288
*MITF*	AD	NM_198159.3	ENST00000352241
*MPZL2*	AR	NM_005797.4	ENST00000278937
*MSRB3*	AR	NM_198080.4	ENST00000355192
*MTAP*	AR	NM_002451.4	ENST00000380172
*MT-RNR1*	Mitochondrial	NC_012920	ENST00000389680
*MT-TL1*	Mitochondrial	NC_012920	ENST00000386347
*MT-TS1*	Mitochondrial	NC_012920	ENST00000387416
*MYH14*	AD	NM_001145809.2	ENST00000601313
*MYH9*	AD	NM_002473.5	ENST00000216181
*MYO15A*	AR	NM_016239.4	ENST00000205890
*MYO3A*	AD, AR	NM_017433.5	ENST00000265944
*MYO6*	AD, AR	NM_004999.4	ENST00000369981
*MYO7A*	AD, AR	NM_000260.4	ENST00000409709
*NARS2*	AR	NM_024678.6	ENST00000281038
*NDP*	X-linked	NM_000266.4	ENST00000378062
*NLRP3*	AD	NM_004895.4	ENST00000336119
*OSBPL2*	AD	NM_144498.3	ENST00000313733
*OTOA*	AR	NM_144672.3	ENST00000388958
*OTOF*	AR	NM_194248.3	ENST00000272371
*OTOG*	AR	NM_001277269.2	ENST00000399391
*OTOGL*	AR	NM_173591.3	ENST00000458043
*P2RX2*	AD	NM_170683.4	ENST00000343948
*PAX3*	AD, AR	NM_181457.4	ENST00000350526
*PCDH15*	AR	NM_033056.4	ENST00000320301
*PDE1C*	AD	NM_001191058.4	ENST00000396193
*PDZD7*	AR	NM_001195263.2	ENST00000619208
*PJVK*	AR	NM_001042702.4	ENST00000409117
*PNPT1*	AR	NM_033109.5	ENST00000447944
*POLR1C*	AR	NM_203290.4	ENST00000372389
*POLR1D*	AR	NM_015972.4	ENST00000399696
*POU3F4*	X-linked	NM_000307.5	ENST00000373200
*POU4F3*	AD	NM_002700.3	ENST00000230732
*PPIP5K2*	AR	NM_001276277.3	ENST00000358359
*PRPS1*	X-linked	NM_002764.4	ENST00000372435
*PTPRQ*	AD, AR	ENST00000614701	ENST00000266688
*RDX*	AR	NM_001260492.1	ENST00000405097
*RIPOR2*	AD, AR	NM_014722.5	ENST00000259698
*S1PR2*	AR	NM_004230.4	ENST00000590320
*SEMA3E*	AD	NM_012431.3	ENST00000307792
*SERPINB6*	AR	NM_004568.5	ENST00000335686
*SIX1*	AD	NM_005982.4	ENST00000247182
*SIX5*	AD	NM_175875.5	ENST00000317578
*SLC17A8*	AD	NM_139319.3	ENST00000323346
*SLC22A4*	AR	NM_003059.3	ENST00000200652
*SLC26A4*	AR	NM_000441.2	ENST00000265715
*SLC7A8*	AD, AR	NM_012244.4	ENST00000316902
*SLITRK6*	AR	NM_032229.3	ENST00000647374
*SMPX*	X-linked	NM_014332.3	ENST00000379494
*SNAI2*	AR	NM_003068.5	ENST00000396822
*SOX10*	AD	NM_006941.4	ENST00000396884
*SSBP1*	AR	NM_003143.3	ENST00000481508
*SYNE4*	AR	NM_001039876.3	ENST00000324444
*TBC1D24*	AD, AR	NM_001199107.2	ENST00000293970
*TCOF1*	AD	NM_001135243.1	ENST00000377797
*TECTA*	AD, AR	NM_005422.2	ENST00000392793
*TMC1*	AD, AR	NM_138691.2	ENST00000297784
*TMEM132E*	AR	NM_001304438.2	ENST00000631683
*TMIE*	AR	NM_147196.2	ENST00000326431
*TMPRSS3*	AR	NM_024022.3	ENST00000291532
*TNC*	AD	NM_002160.4	ENST00000350763
*TPRN*	AR	NM_001128228.3	ENST00000409012
*TRIOBP*	AR	NM_001039141.3	ENST00000406386
*TSPEAR*	AR	NM_144991.3	ENST00000323084
*TWNK*	AD, AR	NM_021830.5	ENST00000311916
*USH1C*	AR	NM_153676.4	ENST00000005226
*USH1G*	AR	NM_173477.5	ENST00000319642
*USH2A*	AR	NM_206933.3	ENST00000307340
*WBP2*	AR	NM_012478.4	ENST00000254806
*WDR92*	AD	NM_138458.4	ENST00000295121
*WFS1*	AD, AR	NM_006005.3	ENST00000226760
*WHRN*	AR	NM_015404.4	ENST00000362057

**Table A2:** Phenotypic and genotypic data of the included patients (AD = autosomal dominant, AR = autosomal recessive, F = female, M = male).

Patient	Included Family Members	Sex	Year of Birth	Severity of Hearing Loss	Genetic Variants Found (All Heterozygous Variants)	Genetic Variants (Protein Level)	Used Panel	Deafness Gene Mode of Inheritance
1	Family 1	M	2011	Moderately severe	*MYO15A* c.3203G > T (class 3) *OTOF* c.4463A > T (class 3) *CDH23* c.2263 C > T (class 3) *DMXL2* c.4937G > A (class 3)	p.(Cys1068Phe) p.(Asp1488Val) p.(His755Tyr) p.(Arg1646Gln)	DOOF_v5_NS DOOF_v5_NS DOOF_v5_NS WESHL panel v2.0	AR AR AR AD
2	Family 2, patient 3 = dizygotic twin	M	2015	Moderate	*GRXCR2* c.182T > C (class 3) *PTPRQ* c.3304C > T (class 3)	p.(Met61Thr) p.(Pro1102Ser)	DOOF_v6.2_NS DOOF_v6.2_NS	AR AR
3	Family 2, patient 2 = dizygotic twin	M	2015	Moderate	*PTPRQ* c.3304C > T (class 3) *TBC1D24* c.169C > T (class 3) *TSPEAR* c.415G > A (class 3)	p.(Pro1102Ser) p.(Arg57Cys) p.(Gly139Ser)	DOOF_v6.2_NS DOOF_v6.2_NS DOOF_v6.2_NS	AR AR AR
4	Family 3, patient 5 = monozygotic twin, patient 6 = sibling	M	2016	Moderate	*OTOGL* c.3400A > G (class 3) *TBC1D24* c.601G > A (class 3)	p.(Ile1134Val) p.(Val201Met)	DOOF_v7_NS	AR AD/AR
5	Family 3, patient 4 = monozygotic twin, patient 6 = sibling	M	2016	Moderate	*OTOGL* c.3400A > G (class 3) *TBC1D24* c.601 G > A (class 3)	p.(Ile1134Val) p.(Val201Met)	DOOF_v7_NS	AR AD/AR
6	Family 3, patient 4 and 5 = siblings	M	2014	Moderate	*OTOGL* c.3400A > G (class 3)	p.(Ile1134Val)	DOOF_v7.1_NS	AR
7	Family 4, patient 8 = sibling	M	2010	Moderately severe	*GIPC3* c.226-1G > T (class 4) *OTOF* c.4981G > A (class 3) *PTPRQ* c.6617G > T (class 3) *SLC17A8* c.1645G > A (class 3) *GIPC3* c.226-1G > T (class 5)	p.(Glu1661Lys) p.(Arg2206Ile) p.(Gly549Arg)	DOOF_v8_NS DOOF_v8_NS DOOF_v8_NS DOOF_v8_NS WESHL panel v2.0	AR AR AR AD AR
8	Family 4, patient 7 = sibling	M	2013	Moderate	*BDP1* c.3364G > A (class 3) *PTPRQ* c.5867A > C (class 3)	p.(Gly1122Arg) p.(Gln1956Pro)	DOOF_v8_NS DOOF_v8_NS	AR AR
9	/	F	2017	Moderate	*SLC26A4* c.1334T > G (class 5) *SLC26A4* c.2234C>T (class 3) *LOXHD1c*.5616C>A (class 3) *MYO15A* c.9493C>T (class 3) *TECTA* c.2725C > T (class 3) *THRAP3* c.2689C > T (class 3)	p.(Leu445Trp) p.(Thr745Met) p.(Asn1872Lys) p.(Arg3165Trp) p.(Arg909Cys) p.(Arg897Trp)	DOOF_v9_NS	AR AR AR AR AD/AR AR
10	/	F	2015	Moderate	*ATP6V0A4* c.2035G > T (class 3) *COL2A1* c.4349T > C (class 3) *CDH23* c.9569C > T (class 3) *MYO7A* c.5866G > A (class 3)	p.(Asp679Tyr) p.(Ile1450Thr) p.(Ala3190Val) p.(Val1956Ile)	DOOF_v7_SYN	AR AD AR AD/AR
11	/	M	2016	Profound	*CDH23* c. 7552G > A (class 3) *COCH* c.644T > C (class 3) *MYO15A* c.9754A > G (class 3)	p.(Val2518Met) p.(Ile215Thr) p.(Asn3252Asp)	DOOF_v8.1_NS	AR AD AR
12	/	F	2011	Profound	*ADCY1* c.1750G > T (class 3) *PJVK* c.839A > C (class 3) *OTOA* c.2654A > G (class 3) *TRIOBP* c.6736G > A (class 3)	p.(Asp584Tyr) p.(Lys280Thr) p.(His885Arg) p.(Glu2246Lys)	DOOF_v4_NS	AR AR AR AR
13	/	M	2017	Moderate	*COCH* c.1075_1076delinsCT (class 3) *RIPOR2* c.2683G > A (class 3) *WFS1* c.1124G > A (class 3)	p.(Ser359Leu) p.(Gly895Ser) p.(Arg375His)	DOOF_v11_NS	AD/AR AR AD/AR
14	/	M	2015	Moderate	*MYO1A* c.277C > T (class 3) *CLIC5* c.991C > T (class 3) *TRIOBP* c.3232C > T (class 3)	p.(Arg93Ter) p.(Arg331Trp) p.(Arg1078Cys)	DOOF_v6_NS	AD AR AR
15	/	F	2018	Moderately severe	*COL11A2* c.4266G > A (class 3) *MTAP* c.-5C > G (class 3) *MYH14* c.2424G > A (class 3) *MYO15A* c.4497G > T (class 3) *MYO3A* c.1559C > T (class 3)	p.(Pro1422Pro) p.(Met808Ile) p.(Gly1499Asp) p.(Ala520Val)	DOOF_v11_NS	AD/AR AR AD AR AR
16	/	M	2016	Moderate	*MYO15A* c.1111C > A (class 3)	p.(Pro371Thr)	DOOF_v7_NS	AR
17	/	M	2018	Mild	*LRTOMT* c.491G > A (class 3) *TECTA* c.4720A > G (class 3) *TRIOBP* c.2776C > T (class 3) *USH2A* c.13709G > A (class 3)	p.(Arg164Gln) p.(Ile1574Val) p.(Arg926Cys) p.(Arg4570His)	DOOF_v10_NS	AR AD/AR AR AR
18	/	F	2009	Mild	No variants found after panel testing		DOOF_v6.2-NS	
19	/	M	2017		*CDH23* c.2341G > A (class 3) *MYH9* c.5234C > T (class 3)	p.(Ala781Thr) p.(Thr1745Met)	DOOF_V8_NS	AR AD
20	/	M	2017	Profound	*GSDME* c.693G > C (class 3) *DMXL2* c.4138G > A (class 3) *OTOF* c.152C > T (class 3) *RDX* c.1696C > T (class 3)	p.(Gnl231His) p.(Ala1380Thr) p.(Pro51Leu) p.(Arg566Ter)	DOOF_v11_NS	AD AD AR AR
21	/	M	2015	Moderately severe	*BDP1* c.566_567dupTC (class 5) *CDH23* c.2192C > T (class 3) *GIPC3* c.83C > A (class 3) *OTOA* c.2654A > G (class 3) *OTOA* c.2971 G > A (class 3) *OTOG* c. 3719C > T (class 3)	p.(Ile190Serfs*11) p.(Thr731Met) p.(Pro28Gln) p.(His885Arg) p.(Glu991Lys) p.(Pro1240Leu)	DOOF_v6_NS	AR AR AR AR AR AR
